# Transferable lessons for care provided to children with intellectual and developmental disabilities based on an analysis of facilitators and barriers to SARS-CoV-2 testing

**DOI:** 10.3389/fped.2024.1449255

**Published:** 2024-11-11

**Authors:** George S. Gotto, Jeriel Bohall, Rachel Northrup, Cheryl Lyn Errichetti, Danielle Chiang, Maureen van Stone, Erin Jones, Megan Meck, Luther Kalb

**Affiliations:** ^1^ Institute for Human Development, University of Missouri Kansas City, Kansas City, MO, United States; ^2^ Maryland Center for Developmental Disabilities, Kennedy Krieger Institute, Baltimore, MD, United States

**Keywords:** SARS-CoV-2 testing, children with IDD, successful treatment models, fuzzy cognitive mapping (FCM), stakeholder engagement

## Abstract

**Introduction:**

The purpose of this article is to report on the lessons learned from parents and caregivers of school-age children with intellectual and developmental disabilities (IDD) in Missouri and Maryland regarding the facilitators and barriers to SARS-COV-2 testing.

**Methods:**

Parents participated in interview sessions that employed fuzzy cognitive mapping (FCM), a reliable knowledge-based method that facilitates democratic discourse to understand how stakeholders make decisions. A total of 94 parents from Missouri (58) and Maryland (36) participated in the FCM sessions.

**Results:**

Eight primary barriers and eight primary facilitating factors were identified that influence a successful SARS-COV-2 test. Analyzing the connections between these factors provided valuable information about not only which ideas were most central to the goal of a successful test, but also which factors could be modified to improve the likelihood of success. Results indicate that the physical environment and child preparedness play a central role in successful SARS-COV-2 testing for children with IDD; however, these factors within the context of other invasive procedures should be studied further.

**Discussion:**

It is likely that the findings from this study are transferable to other diagnostic procedures such as influenza, respiratory syncytial virus (RSV), and methicillin-resistant Staphylococcus aureas (MRSA), which require similar testing techniques using a nasopharyngeal swab.

## 1 Introduction

Recent estimates indicate that almost 18% of children have a developmental disability in the United States, and 1.2% of all US children have an intellectual disability (1). As a group, individuals with IDD have far worse health outcomes than those without IDD (2). For example, people with disabilities report higher rates of chronic conditions than those without disabilities, such as obesity, diabetes, and cardiovascular disease (2). Despite co-occurring, complex medical conditions, individuals with IDD are less likely to receive preventive healthcare services (3, 4).

There are a variety of barriers that contribute to this disparity in children with IDD. Cognitive and communication difficulties may make it difficult for children with IDD to understand the rationale for medical procedures or to request accommodations from providers (5). Sensory issues and co-occurring psychiatric disorders such as anxiety may also cause distress for children with IDD in healthcare settings (5). Individuals with disabilities also report structural-environmental barriers to healthcare, including ramp unavailability and inaccessible examination rooms and equipment in healthcare settings (6). Process barriers such as a lack of disability knowledge and sensitivity from providers, as well as insufficient time to address one's needs have also been cited by individuals with disabilities (6). The comorbidities associated with IDD and the barriers to healthcare for children with IDD contribute to an ongoing cycle of unmet healthcare needs and risks for this vulnerable population.

Comorbidities and barriers to effective healthcare placed individuals with IDD at a greater risk for severe SARS-COV-2 outcomes compared to those without IDD. Children with IDD are also at a higher risk of SARS-COV-2 infection due to challenges adhering to mitigation strategies. Sensory issues that are common in children with IDD may make it more challenging for them wear a mask. Additionally, social distancing is more difficult for many children with IDD, especially those with complex medical conditions. These children often require assistance with activities of daily living such as eating, during which mask wearing and social distancing cannot occur. In a study of over 64 million patients, people with IDD were more than twice as likely to be hospitalized if diagnosed with SARS-COV-2 than people without IDD (7). Another study found that among children under 17, those with IDD had higher SARS-COV-2 fatality rates (1.6%) than those without IDD (0.1%) (8).

Beyond the direct risks posed by the SARS-CoV-2 virus itself, the pandemic negatively impacted children with IDD and their families in a variety of ways. Children with IDD rely on schools for various services beyond education, including speech, occupational, and physical therapy, social services, and psychological interventions. According to an online survey for caregivers of individuals with IDD, 78% reported that their child lost access to at least one therapy or educational service due to SARS-COV-2 restrictions (9). In addition, Chafouleas and Iovino (10) found that during the pandemic, caregivers of children with disabilities reported significantly higher levels of burden, stress, anxiety, and depression than caregivers of typically developing children. It is abundantly clear that children with IDD are at a high risk for a variety of negative outcomes related to the SARS-COV-2 pandemic.

SARS-CoV-2 testing is a prevention strategy recommended by the Centers for Disease Control and Prevention (CDC) to promote the health and safety of children and their families. Since children with IDDs are disproportionately impacted by SARS-COV-2, access to testing is especially important. However, like other preventive health measures, various barriers interfere with SARS-COV-2 testing for this population. Yet, research surrounding barriers and facilitators to SARS-COV-2 testing for children with IDD is limited. To the authors’ knowledge, Haroz and colleagues (11) were the first to examine barriers to SARS-COV-2 testing in a variety of underserved pediatric populations. However, this study was limited to school-based testing and only examined the perspectives of the research teams, rather than key stakeholders such as the caregivers of the children being tested. The current study aims to address this gap in the literature by assessing how caregivers perceive facilitators and barriers to SARS-COV-2 testing for children with IDD, utilizing a participatory methodology called Fuzzy Cognitive Mapping (FCM). Finally, we argue that the lessons learned from this research are transferrable to multiple settings and diagnostic procedures.

## 2 Method

### 2.1 Fuzzy cognitive mapping

Fuzzy Cognitive Mapping (FCM) was employed to identify the facilitating factors and barriers to SARS-COV-2 testing among school-age children with IDD. Axelrod (12), a political scientist, was one of the first to use FCM as a means of capturing relational data in a logical and visual format. Specifically, he used it to represent social scientific knowledge using nodes (variable concepts) and edges (causal connections). Later, Kosko and colleagues (13) used FCM to structure virtual worlds (14) and map policy scenarios (15). More recently, social scientists have used FCM to gather the viewpoints that influence decision and sense-making across multiple stakeholders (16–19). These projects have shown FCM to be a reliable knowledge-based model that facilitates democratic discourse to understand how stakeholders make decisions (20). This methodology is highly participatory and fosters social learning between participants. The original purpose for hosting FCM sessions for the present project was to generate community-specific, testable strategies to address social factors that impact the uptake and effectiveness of SARS-COV-2 testing, which will be referred to as “COVID-19 testing” throughout the discussion below.

### 2.2 Sample

A total of 94 people participated in mapping sessions across Maryland (*n* = 36) and Missouri (*n* = 58). The majority were biological mothers of a child with IDD (74.5%). Participants’ ages ranged from 25 to 64 (*M* = 44.6), 89% identified as female, 75% identified as White, 67% were married or partnered, 52% reported working full-time, 82% had post-secondary and higher education, and 22.3% lived in a rural setting (see Table 1). Over 75% of the participants had at least one child with IDD enrolled in school. The participants reported that their children had co-occurring disabilities, with the most common being autism (76%) followed by developmental delay (40.1%), intellectual disability (33.6%), and speech or language impairment (33.5%).

**Table 1:** Participant demographic information: overall participants (*N* = 94).

Demographic categories	Missouri		Maryland		Total	
	*n*	%	*n*	%	*N*	%
Age in years (*M* = 44.6)						
25–30	3	5.2%	0	0.0%	3	3.2%
30–39	15	25.9%	6	16.7%	21	22.3%
40–49	26	44.8%	17	47.2%	43	45.7%
50–59	10	17.2%	9	25.0%	19	20.2%
60–64	2	3.4%	4	11.1%	6	6.4%
Other or missing	2	3.4%	0	0.0	2	2.1%
Gender						
Female	54	93.1%	30	83.3%	84	89%
Male	3	5.2%	6	16.7%	9	9%
Other or missing	1	1.7%	0	0.0%	1	1.0%
Race						
White	47	81.0%	23	63.9%	70	74.5%
Black or African American	6	10.3%	9	25.0%	15	16.0%
Asian	1	1.7%	2	5.6%	3	3.2%
Two or more races	2	3.4%	1	2.8%	3	3.2%
Other or missing	2	3.4%	1	2.8%	3	3.2%
Ethnicity						
Hispanic	2	3.4%	0	0.0%	2	2.1%
Non-Hispanic	55	94.8%	35	97.2%	90	95.7%
Other or missing	1	1.7%	1	2.7%	2	2.1%
Marital status						
Married	27	46.6%	30	83.3%	57	60.6%
Divorced	12	20.7%	3	8.3%	15	16.0%
Separated	2	3.4%	1	2.8%	3	3.2%
Single	7	12.1%	0	0.0%	7	7.4%
Widowed	2	3.4%	0	0.0%	2	2.1%
Living with someone but not married	6	10.3%	0	0.0%	6	6.4%
Other or missing	2	3.4%	2	5.6%	4	4.3%
Employment						
Full time	29	50.0%	20	55.6%	49	52.1%
Part time	7	12.1%	7	19.4%	14	14.9%
Home maker or stay-at-home caregiver	13	22.4%	5	13.9%	18	19.1%
Retired	1	1.7%	2	5.6%	3	3.2%
Unemployed	1	1.7%	2	5.6%	1	1.1%
Other or missing	7	12.1%	20	55.6%	9	9.6%
Highest level of education						
Doctorate degree	3	5.2%	4	11.1%	7	7.4%
Master's degree	14	24.1%	14	38.8%	28	29.8%
Bachelor's degree	16	27.6%	13	36.1%	29	30.9%
Associates degree	10	17.2%	3	8.3%	13	13.8%
Some college	5	8.6%	2	5.5%	7	7.4%
High school/GED	6	10.3%	0	0.0%	6	6.4%
Annual household income						
$100,000–150,000	6	10.3%	9	25.0%	15	16.0%
$75,000–99,999	9	15.5%	3	8.3%	12	12.8%
$50,000–74,999	13	22.4%	1	2.8%	14	14.9%
$20,000–49,999	17	29.3%	2	5.6%	19	20.2%
Less than $20,000	8	13.8%	1	2.8%	9	9.6%
More than $150,000	1	1.7%	14	38.9%	15	16.0%
Other or missing	4	6.9%	6	16.7%	10	10.6%
Setting						
Rural	17	29.3%	4	11.1%	21	22.3%
Urban	9	15.5%	8	22.2%	17	18.1%
Suburban	28	48.3%	22	61.1%	50	53.2%
Other or missing	4	6.9%	2	5.5%	6	6.4%
Parent child relationship						
Biological mother	42	72.4%	28	77.8%	70	74.5%
Biological father	3	5.2%	3	8.3%	6	6.4%
Adoptive mother	7	12.1%	4	11.1%	11	11.7%
Adoptive father	0	0%	1	2.8%	1	1.1%
Other or missing	6	10.3%	0	0.0%	6	6.4%
Number of children with disabilities enrolled in school						
1	41	70.7%	33	91.7%	74	78.7%
2	12	20.7%	2	5.6%	14	14.9%
3	1	1.7%	1	2.8%	2	2.1%
Other or missing	4	6.9%	0	0.0%	4	4.3%

#### 2.2.1 Data collection

**Figure 1:**
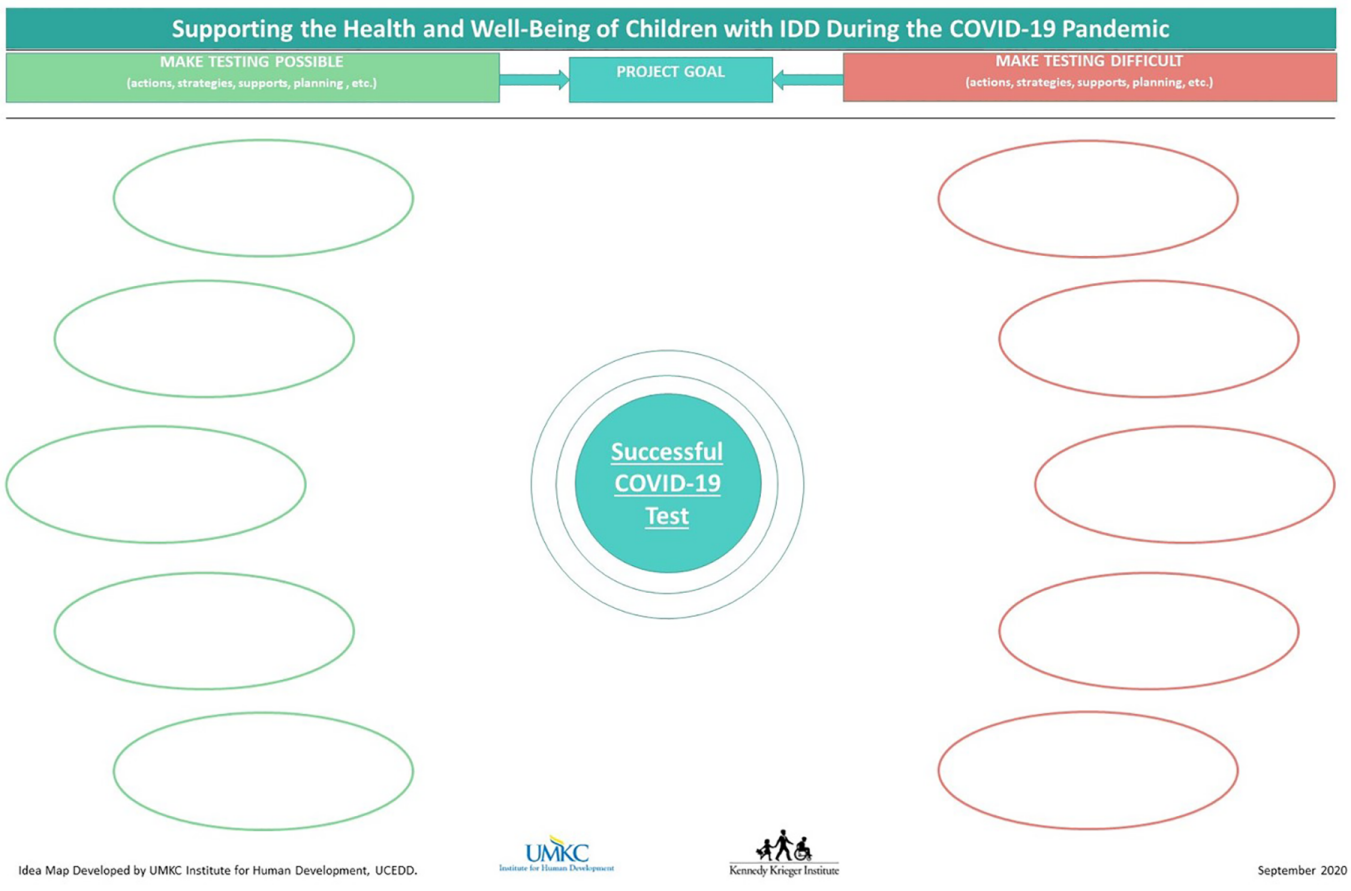
Blank fuzzy cognitive mapping template.

The FCM sessions were organized at the community level in partnership with Missouri Family-to-Family and the Kennedy Krieger School Programs. Leadership from partnering organizations worked with the team to recruit parents/guardians who were representative of the gender, ethnic/racial, and social/economic diversity in Maryland and Missouri. Due to the pandemic, FCM sessions took place in an online Zoom format using a program called Draw Chat. This whiteboard program allows collaborators to work on a document in real time via audio and video conferencing systems (e.g., Zoom). Each participant had a blank template on which to draw their maps (see Figure 1). The sessions were capped at 10 participants, but most sessions only included three to six participants.

The research team provided written instructions and a tutorial for all participants. In each FCM session, participants were asked to list up to five important facilitators and five barriers to successful SARS-COV-2 testing among children with IDD using the blank template (Figure 1). Participants then applied directional lines (arrows) depicting the perceived connections between all concepts listed on their maps. Next, they weighted the connections (i.e., 1 = mild connection; 2 = moderate connection; 3 = strong connection) to quantify the relationships between the items on their maps. The research team then facilitated group discussion of the ideas presented on the maps. Conversations during the FCM sessions were audio recorded to provide context to the data on the maps. A minimum of two members of the research team were present for each FCM session.

#### 2.2.2 Data cleaning and preparation

The first step in the data cleaning and preparation process was to conduct an item level analysis of each map (21). Eight members of the research team read each map individually and then developed a codebook (Table 2). This process involved discussing the contents of the maps, identifying code names, and defining codes until a consensus was reached. Ultimately, eight broad facilitator concepts used by the participants and eight barrier concepts were identified.

**Table 2:** Codebook with definitions.

Codes	Definition
Facilitators	
Preparing the child	Explaining testing process, modeling, social story, practicing, touch testing materials, advance notice, etc.
Parent/guardian involvement	Role the parent wants to play in the testing (i.e., administer the test, restrain, comfort the child)
Good environment	Environment of test is accommodating of different needs, privacy options, number of people, a place the child is familiar with, accessible, familiar support person, etc.
Knowledgeable test administrator	Culturally competent, understanding needs of child with disabilities, compassionate/patient, person-centered practitioner.
Distraction	Bubbles, iPad, fidget toy, comfort item, headphones, etc.
Ease of logistics	Ease of signing up/rescheduling, time of test, testing site, easy to navigate, distance to testing site, etc.
Mode of test (painless)	Swab, saliva, results timing, description of test experience, self-test, etc.
Incentives/motivators/reinforcers	Reward for taking the test, praise, stickers, food, special toy, etc.
Barriers	
Poor environment	Too much stimuli, bright lights, too many people, wait time, being exposed while testing, safety concerns, lack of supports, unfamiliar place, etc.
Poor test administrator	Tired/grumpy/unprepared healthcare provider, impatient, does not understand unique needs of the child
Costs to family	Financial and emotional costs. Transportation cost, lost wages/time off cost, cost of test, childcare costs for other children, stress, etc.
Child needs	Child needs that can make testing difficult, personality, age, mood, negative feelings about the test, behaviors, etc.
Mode of test (painful/invasive)	Swab, saliva, results timing, description of test experience, self-test, etc.
Inconvenient logistics	Ease of scheduling, distance to testing site, location of test, access to information, etc.
Frequency of test	Having to do the test more than once, negative experience influences the next one, concerns for daily testing, etc.
Stigma	What is said on the news, social media, by peers about COVID19/testing; negative stigma for positive test.

### 2.3 Codebook

The same team members then broke into four teams of two researchers. Each team was randomly assigned 24 maps. When a team finished coding their maps, another team coded the same maps without seeing the first team's coding structure. Ultimately, every map was coded independently by each team. The four teams came together weekly to discuss their observations and any questions that arose during the coding process. Finally, the four teams came together to discuss any areas of disagreement on each map until agreement levels reached 100%.

#### 2.3.1 Data analysis

Once the maps were coded, Excel was used to create an adjacency matrix for each map based on the directional and weighted connections identified by the participants. The 94 matrices were then merged into one dataset and exported to other software for final analysis. Specifically, FCMapper, an Excel-based program, was employed to analyze the mapping data using matrix algebra tools of graph theory and to conduct simulation or “scenario” testing. Next, cognitive modeling was conducted using Pajek, a program that operates much like a scientific calculator to visually explore network objects. Using a partitioning feature within this program, variables (or nodes) could be classified within the network by degree of input and output, and subsequently, the graph could be layered to assign nodes with similar values to the same plane. Additionally, by using vectors, the centrality values of each variable could be factored into the overall layout of the model. These centrality values determine the proximity, or “betweenness” of nodes on the model. Finally, structural equation modeling (SEM) was used to confirm the results of the FCM analyses. SEM is a combination of confirmatory factor analysis (CFA) and multiple regression (22) that identifies the relationships among latent constructs. For the purpose of the current study, SEM analyses examined the effects of facilitators and barriers related to a Successful SARS-COV-2 Test.

## 3 Results

### 3.1 Graph theory

The initial analysis, which employed FCMapper, analyzed the mapping data using matrix algebra tools of graph theory. This allowed the structure of the maps to be examined to determine how participants viewed the actions, strategies, or supports that impact SARS-COV-2 testing. There were 17 variables and 107 connections, and the density index was 0.37. Density is an index of map connectivity, and it is calculated by dividing the number of connections by the maximum number of connections possible between *N* variables [*D* = *C*/*N*^2^; (23)]. For the present sample, the total number of possible connections between the 17 variables was 289 connections. Another way to interpret the density score is to recognize that the 107 connections used by our participants only represent 37% of the total possible connections. The participants’ maps were not highly complex but they were largely in agreement about how variables impacted a successful SARS-COV-2 test.

Within FCM methodology, there are three types of variables: receiver, transmitter, and ordinary. The variable type is important because it shows how the variables act in relation to one another [(24), p. 51]. Receiver concepts are those that are only affected by other system concepts and have no effect (or output) on other concepts. Participants in the current study were provided with the end point, or goal, for their maps: Successful COVID-19 test. This was the leading receiver variable, as expected, because all other concepts had either direct or indirect influence on the goal. As it turns out, “Successful COVID-19 Test” was the only receiver concept within the data. There were no transmitter concepts resulting from the data, which are variables that only impact others but do not receive input from other variables. Therefore, the remaining 16 variables were ordinary, meaning they played both a transmitting and a receiving function—they both influenced and were influenced by other concepts.

Finally, the centrality of each variable was calculated. Centrality shows how connected a variable is to other variables and the cumulative strength/weights of those connections. In FCM methodology, a variable can be more “central” if the connections to that variable are strong, even though it might have fewer connections altogether (13). As described by Özesmi & Özesmi (24), centrality is “not only a frequency of expression but also how important that variable is given the whole structure of the cognitive map.” As expected, the goal (Successful COVID-19 Test) had the highest centrality value (3.28) since it was the ultimate endpoint for the other concepts. The variables with the highest centrality values from there were: 1. Preparing the child (.86); 2. Poor environment (0.65); 3. Good environment (0.64); and 4. Child Needs (0.62). If the two environmental variables are combined, it is apparent that the testing environment- including both positive and negative influences - is highly central to SARS-COV-2 testing among children with IDD. Of note, variables with the lowest centrality values (least impactful to a successful SARS-COV-2 test) were “Costs to the Family” (0.05), “Stigma” (0.06), and “Frequency of Test” (0.11).

### 3.2 Cognitive modeling

**Figure 2:**
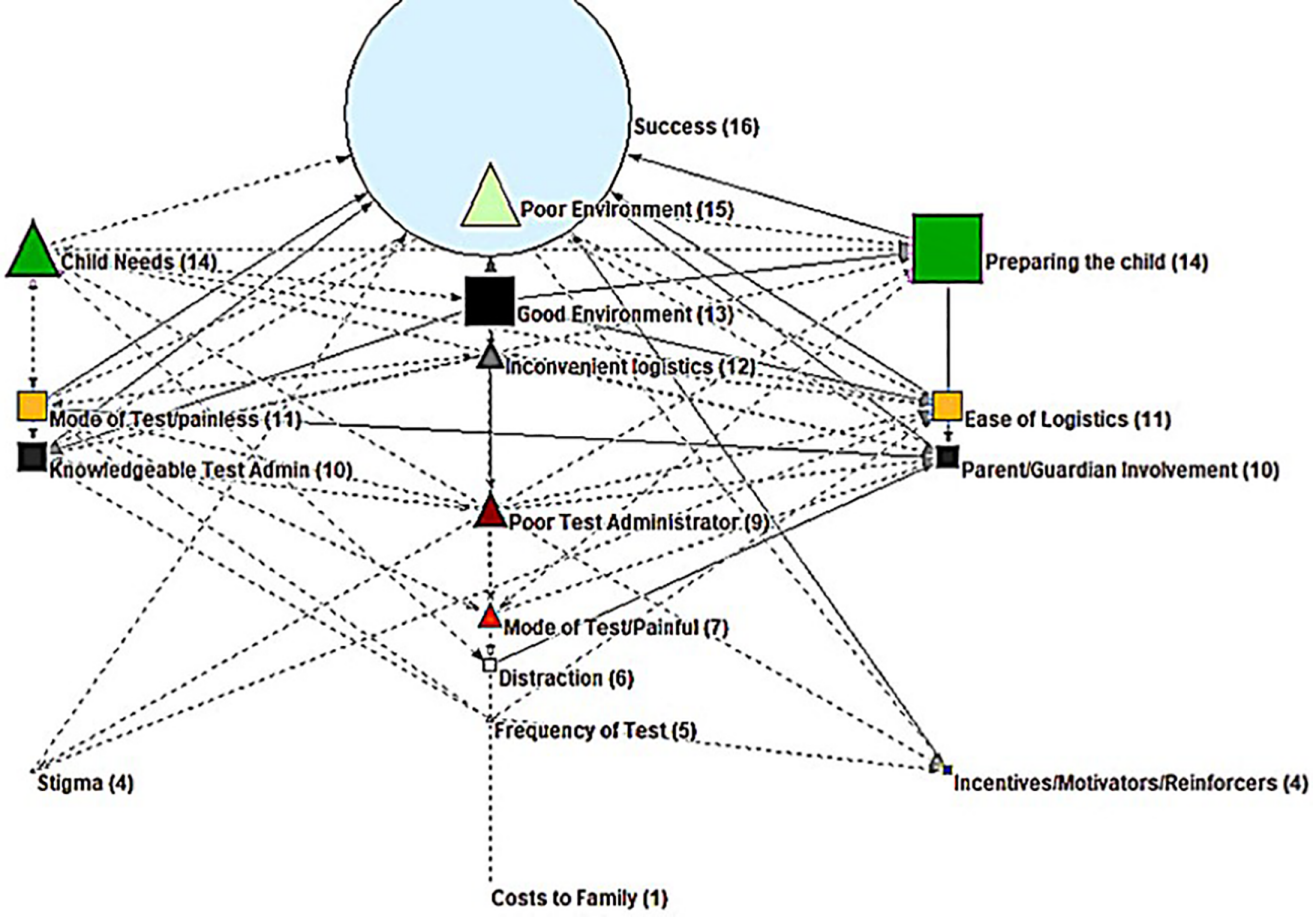
Synthesized and modeled FCM.

As mentioned above, Pajek was used to visually graph FCM data to display each variable and its connections within the system. Figure 2 shows the cognitive model with all variables (facilitators and barriers) and their connections. The nodes represent the 17 codes used when building the FCM matrix, and the arrows between them represent the directional relationships among these concepts. Facilitating concepts are displayed as squares, and barrier concepts are displayed as triangles. In most cases, participants connected facilitators to the end goal “Successful COVID-19 Test,” whereas the barriers had additional connections to one or more facilitators. In these cases, it can be understood that barriers not only inhibited the goal directly, but also inhibited facilitating factors increasing their total negative impact on the goal.

The size of the nodes represents the impact on the overall model, with larger shapes indicating greater impact and smaller shapes indicating weaker impact. The most impactful facilitator, corresponding with the highest centrality, was “Preparing the Child.” This concept included ideas such as: providing a social story or video of the procedure; imparting knowledge that the test is coming; ensuring that the child gets a good night's rest before the test; and explaining why the test is being performed. The second most impactful facilitator was “Good Environment,” which included ideas such as: minimal waiting times; quieter setting; sensory-friendly environment; and comforting the child. The facilitator concept “Knowledgeable Test Administrator” related to having a calm, flexible person to administer the test; having positive, smiling, and encouraging people to administer; having a patient tester who could slowly explain what will happen as many times as needed; and other factors specific to the person administering the test. Another impactful facilitator was “Mode of Test: Painless,” which included ideas such a painless/non invasive test and using a saliva or alternative test instead of a nasal swab.

The most impactful barrier to successful SARS-COV-2 testing for children with developmental disabilities was “Poor Environment.” This can be seen near the top of the model on the highest “layer,” and it included concepts such as: long wait times; not having available behavioral support; chaotic or busy testing environment; sensory overload problems, etc. The second most impactful barrier was “Child Needs” and included concepts such as: inherent difficulties associated with special needs children (such as mobility); child age and/or challenging personality traits; not having advanced notice about the test; difficulties restraining the child, anxiety, and behavioral meltdowns. Another significant barrier, “Inconvenient Logistics,” which included concepts such as distance to the testing site and its location, access to information, and ease of scheduling.

### 3.3 Structural equation modeling (SEM) results

Finally, structural equation modeling was used to confirm the results of the graph theory and cognitive modeling. There were 16 concepts whose impact on “Successful COVID 19 Test” was estimated. As we mentioned above, eight were facilitators concepts: Preparing the Child (*n* = 71), Good Environment (*n* = 61), Mode of Test: painless (*n* = 40), Knowledgeable Test Administrator (*n* = 38), Ease of Logistics (*n* = 38), Incentive/Motivators (*n* = 32), Distraction (*n* = 30), Parent/Guardian Involvement (*n* = 29). There were also eight barriers: Child Needs (*n* = 65), Poor Environment (*n* = 65), Poor Test Administrator (*n* = 53), Mode of Test: painful/invasive (*n* = 44), Inconvenient Logistics (*n* = 37), Frequency of Test (*n* = 12), Costs to Family (*n* = 10), and Stigma (*n* = 5).

#### 3.3.1 One factor model

**Figure 3:**
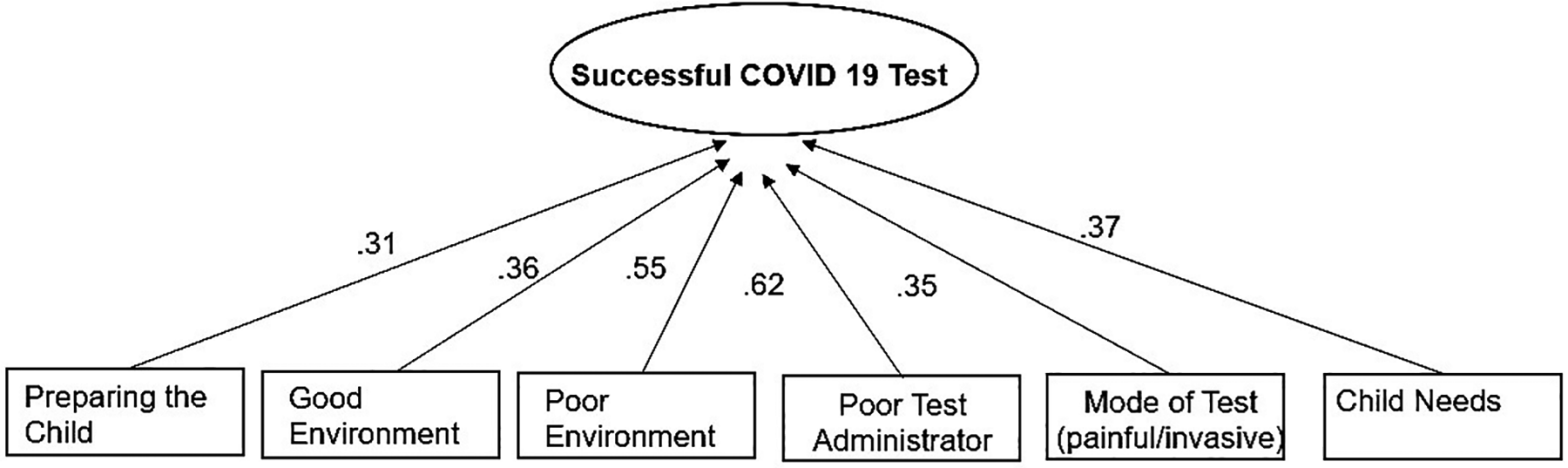
One factor SEM model.

In the one-factor model, SEM was used for pinpointing key items that loaded onto Successful SARS-COV-2 Test. Based on the global model fit indices and the factor loadings in each model, variables with the least factor loading (smaller than.3) were removed from the model until acceptable model fit was achieved. Six items remained in the final model. The one-factor model had a good fit: *χ*^2^ (74) = 75.89, *p* = .42, CFI = .97, TLI = .97, RMSEA = .02, SRMR = .06. The path diagram of the structure with the six variables are shown in Figure 3. Results indicated “Preparing the Child,” “Good Environment” “Poor Environment,” “Poor Test Administrator,” “Mode of Test: painful/invasive,” and “Child Needs” had a direct effect on Successful SARS-COV-2 Test and thus can be deemed most impactful variables. The SEM results are congruent with the FCM results.

#### 3.3.2 Two-Factor model

**Figure 4:**
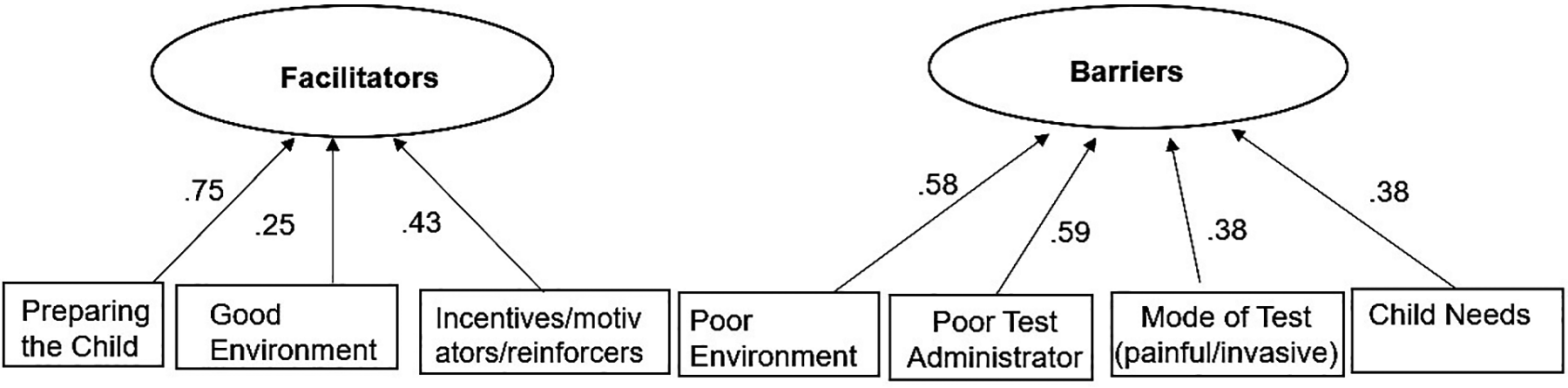
Two factor SEM model.

In the two-factor model, SEM was used to pinpoint key items that load onto Facilitators and Barriers. Both Facilitators and Barriers were latent variables in this model. The two-factor model had a good fit: *χ*^2^ (73) = 68.72, *p* = .62, CFI = 1.00, TLI = 1.00, RMSEA < .001, SRMR = .06. Results indicated Preparing the Child, Good Environment, Incentives/Motivators have a direct effect on Facilitators (Figure 4). Poor Environment, Poor Test Administrator, Mode of Test: painful/invasive and Child Needs have a direct effect on Barriers.

Table 3 presents the item level estimates and factor loadings below. The one-factor loadings range from .31 to.62, and the two-factor loadings ranged from 0.38 to 0.75 with significant *p*-values.

**Table 3:** Factor loadings (*N* = 94).

	One factor loadings		Two factor loadings	
	Effect (*λ*)	SE	Effect (λ)	SE
Facilitators				
Preparing the child	0.31	0.14	0.75	0.23
Good environment	0.36	0.14	0.25	0.15
Incentives/motivators	-	-	0.43	0.13
Barriers				
Poor environment	0.55	0.14	0.58	0.18
Poor test administrator	0.62	0.14	0.59	0.16
Mode of test (painful/invasive)	0.35	0.14	0.38	0.15
Child needs	0.37	0.14	0.38	0.17

## 4 Discussion

We used FCM to identify stakeholder-generated, testable strategies for successfully conducting SARS-COV-2 tests among children with IDD. The SARS-COV-2 pandemic presented many challenges and a significant disruption to the routines of daily life, especially for individuals with IDD. Testing children with IDD for SARS-COV-2 carries many considerations that healthcare workers and other test administrators may not be accustomed to. Without specialized training, testing sites may be poorly prepared to handle the physical, behavioral, and other unique needs of children with IDD. This bears with it the risk of unsuccessful testing and potentially misdiagnosed or underdiagnosed cases of SARS-COV-2 in this population. Using the FCM methodology to collect data from families of children with IDD, who are key stakeholders in the testing process, provided valuable information about not only which ideas were most central to the goal of a successful test, but also which factors could be modified to improve the likelihood of success.

### 4.1 Recommendations

Results showed that the environment plays a central role in the testing experience for children with IDD, including both positive and negative environmental factors. The facilitator “good environment” and the barrier “poor environment” together had the highest centrality in the model. Translating these findings to practice, it is recommended that facilitating SARS-COV-2 testing in children with IDD should initially be aimed at environmental factors. Based on feedback received on the original concept maps reviewed, improving environmental efforts could include trying to minimize wait times for children with IDD, facilitating a quiet environment, promoting a culture of comfort, and producing sensory-friendly testing areas. Ideas for promoting a sensory-friendly environment might include modifying the color or intensity of light sources, reducing “visual clutter,” and offering headphones to children to help reduce ambient noise – all of which are customizable depending on the child's needs (25). Other testing strategies have been developed specifically for students and staff in schools for children with IDD and complex medical conditions. A report by Sherby and colleagues (26) describes three research teams’ approaches to testing in various specialized school programs. These teams implemented a variety of techniques, keeping in mind the unique needs of children with IDD. For instance, investigators at Washington University in St. Louis developed a testing procedure that involved collecting a small quantity of saliva, rather than the invasive nasopharyngeal swab procedure utilized by many other SARS-COV-2 testing sites (26). Investigators at the University of Wisconsin-Madison were able to implement in-home testing for school-aged children with complex medical conditions, allowing for parents to conduct the tests on their child (26). Similar to the research described in this article, these projects aim to identify generalizable testing models to increase successful healthcare provision to children with IDD and co-occurring medical conditions.

Preparing the child for the test was also identified as an important facilitator. Ideas for preparing the child, as indicated by the participants, might include providing a social story or video of the procedure, explaining to the child that the test is coming and why it is important, and educating caregivers about the importance of a good night's sleep prior to testing. This finding echoes Haroz and colleagues (11) who recommend identifying and preparing key stakeholders involved in testing. They found that one of the biggest challenges to implementing SARS-COV-2 testing was an inability to engage relevant stakeholders [e.g., families, students, and staff; (11)].

The relationships represented within the FCM model, indicate that making suggested changes may result in improvements in other areas. For example, the FCM model indicates that the “mode of test (painful)” barrier is impacted when environment was altered. Previous evidence suggests that a child who is calm vs. anxious is likely to experience less pain (27). It is also reasonable to expect that a calm, conducive environment will facilitate parent or caregiver involvement, in addition to the confidence and effectiveness of the test administrator.

The barrier “Child Needs” was another central concept within the model. This variable is challenging to address in many ways, since the physical and behavioral needs for children with special needs can vary dramatically. However, the FCM model indicates that increasing child preparedness will lessen the impact of the child's disability-specific needs. This is possibly explained by the extra time spent by test administrators to talk to the child, explain the procedure, allowing them to examine testing implements, which allows test administrators the opportunity to establish a positive social relationship with the child and assess for unique needs. Knowing their needs beforehand and adequately preparing the child, test administrators could better prevent behavioral incidences, reduce anxiety, and avoid restraining procedures. Mitigating child needs through other variables, rather than directly trying to change disability-related needs, is something worth considering.

#### 4.1.1 Transferable lessons

While the scope of this study only models SARS-COV-2 testing in children with IDD, there may be value in extending these recommendations to other testing procedures within this population. Results indicate that environment and child preparedness play a central role in successful SARS-COV-2 testing for children with IDD; however, these factors within the context of other invasive procedures should be studied further as it is likely that the findings from this study are transferable to other diagnostic procedures. For example, many other infections such as influenza, respiratory syncytial virus (RSV), and methicillin-resistant Staphylococcus aureas (MRSA) require similar testing techniques using a nasopharyngeal swab. Testing for Strepotococcus (strep throat) involves a throat swab, which may be a similarly distressing experience to children with IDD. Opportunities to improve procedural experiences for children with IDD could even be extended to areas such as: needle sticks for vaccines, medication administration, and blood draws, blood pressure checks and application of monitoring devices, care and dressing of wounds, dental and vision procedures, fitting for orthopedic devices, and others. It is likely that improving child preparedness and the environmental space in which these procedures are performed could have far-reaching impact within a variety of settings, including medical offices, school nursing offices, dentistry, therapy centers, stand-alone testing sites, and even home-based health services. Thus, it is worth considering how these recommendations could be applied in the daily practice of any healthcare professional, including therapists, dentistry specialists, school nurses, nursing assistants, laboratory and imaging personnel, and even volunteers.

Altering the environment and taking the time to prepare a child may seem like daunting tasks in fast-paced healthcare settings that already struggle to manage patient loads. If so, it may be beneficial for clinical leaders to help staff identify and start with small, manageable changes, such as dimming light sources or turning on calming music as well as allowing a known caregiver to attend. Additionally, it is helpful to remember that the extra time it might take to prepare a child for a procedure is not only important for that child's well-being, but it can also prevent challenging and time-consuming situations resulting from behavioral crises.

### 4.2 Limitations

While FCM methodology provides an organized, structured process for analyzing qualitative data, there is the potential for bias to be introduced while coding qualitative data for emerging themes. Steps were taken to minimize this bias by dividing cognitive maps between teams, coding them separately, and then meeting together to discuss coding results and areas of disagreement. Additionally, although strong correlations were identified between certain concepts and the goal of a successful SARS-COV-2 test, this study did not specifically evaluate different interventions that could enhance these concepts. The impact of specific environmental changes on children with IDD within the testing area, as one example, should be studied further.

The race and ethnicity of the samples in both Missouri and Maryland were not fully representative of the state populations in 2021, when data were collected. For example, in Missouri, White residents were 77% of the state population but they made up 81% of the Missouri sample. Black or African American participants (10.3% sample, 11.4% state) and Hispanic participants (3.4% sample, 4.9% state) were slightly underrepresented (28). In Maryland, 25% of the sample identified as Black or African American, slightly under the statewide population statistics (29.5%). Hispanic residents were not represented in the Maryland sample even though 11.8% of Maryland's population was Hispanic. The percentage of White study participants in Maryland (63.9%) was over representative of the statewide population [48.7%; (29)]. Other races and ethnicities, including Asian and American Indian and Alaska Natives, were underrepresented in both states.

## 5 Conclusions

As impacts from the SARS-COV-2 pandemic linger, new variants of the virus emerge, and new infection control recommendations are developed, testing children with IDD needs to be a viable option for proper diagnosis and treatment. Children with IDD are especially vulnerable to the effects of the pandemic, and this population brings many unique needs that may be challenging for testing sites to manage. The FCM process helped evaluate both facilitating and inhibiting variables surrounding successful SARS-COV-2 testing from the perspective of key stakeholders. This provided valuable insight into which of these variables are most central to success and where efforts can be applied to leverage the highest improvement potential.

The FCM model revealed that environmental factors, preparing the child for the test, and child needs were the most central concepts surrounding successful testing. Other important variables included logistics, mode of test (painless), knowledgeable test administrators, and parent/guardian involvement. Costs to family, stigma, and frequency of the test were least central. Through scenario testing, it could be seen that environmental changes had a positive influence on preparing the child for the test as well as the goal of a successful test. Additionally, improving child preparedness by itself had a positive impact on the overall model. The best result obtained through scenario testing, however, was when environment and child preparedness were improved simultaneously. This indicates that to achieve a successful SARS-COV-2 test in children with IDD, efforts should be focused on both environmental modifications as well as steps to prepare the child for the test.

## Data Availability

The raw data supporting the conclusions of this article will be made available by the authors, without undue reservation.
